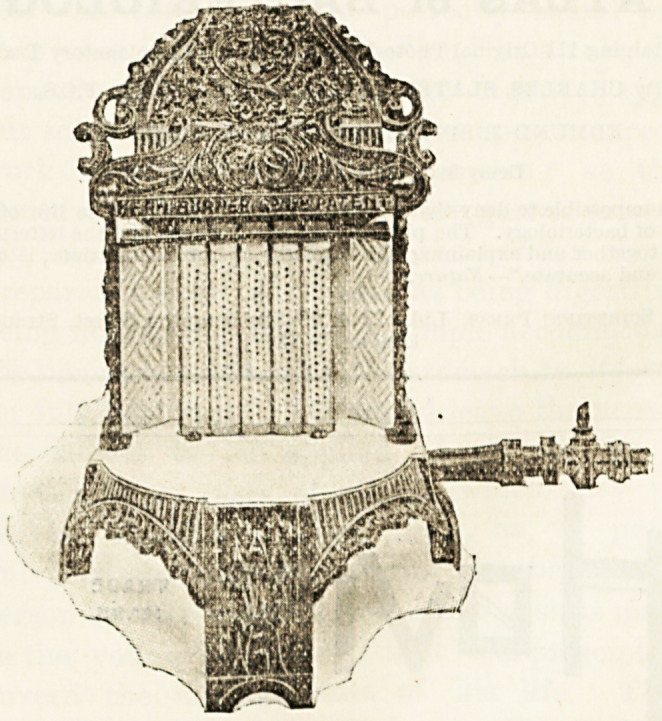# Practical Departments

**Published:** 1901-10-26

**Authors:** 


### PRACTICAL DEPARTMENTS.

### A NEW DEVELOPMENT IN HEATING BY GAS.

Two special defects are frequently brought against gas stoves which have hitherto mitigated against their general adoption, in spite of the great convenience which they can justly claim. These defects are vitiation of air, and expense. The Kern Burner Company have just introduced a stove of

French parentage, called the Clamond stove, which has neither of these disadvantages, and which therefore should introduce a new era in the use of gas for warming purposes. The Clamond gas stove gives a powerful radiant heat. The burners are each fitted with a small upright clay pipe, through which the gas is supplied, and burns with the exact amount of mixed air and gas as to ensure perfect combustion, with the result that, so far as the carbon and the hydrogen in the gas are concerned, nothing is produced except pure watery vapour and carbonic acid gas. The heating capacity of this principle is so great that a small stove, burning only 10 feet of gas per hour, is sufficient to heat a medium-sized room, with a cost of a little over one penny for three hours. A slightly larger stove has the immense advantage of two taps, so that only half the number of burners can be used when desired. In ordinary gas stoves, the lowering of the gas to reduce heat causes imperfect combustion. The Clamond stoves are small and of most elegant design. They are inexpensive, and may be placed anywhere most convenient, as they do not require a connection with a chimney, though one can be used if preferred. Where the connection with a chimney is dispensed with, a trivet is fixed upon the stove, a very useful adjunct, which is supplied free of extra cost. The Kern Burner Company, Limited, Gravel Lane, Southwark, London, S.E., has a large showroom where their admirable stoves are on view, and they also issue an illustrated catalogue. The Clamond method can be adapted for use in an ordinary fireplace, or existing gas stove.

## Figures and Tables

**Figure f1:**